# Repurposing an EMG Biofeedback Device for Gait Rehabilitation: Development, Validity and Reliability

**DOI:** 10.3390/ijerph18126460

**Published:** 2021-06-15

**Authors:** Reza Koiler, Elham Bakhshipour, Joseph Glutting, Amy Lalime, Dexter Kofa, Nancy Getchell

**Affiliations:** 1 Biomechanics and Movement Science Interdisciplinary Program, University of Delaware, Newark, DE 19716, USA; elhambak@udel.edu (E.B.); getchell@udel.edu (N.G.); 2 School of Education, University of Delaware, Newark, DE 19716, USA; glutting@udel.edu; 3 Product & Marketing Manager, mTrigger, LLC, Newark, DE 19713, USA; amyl@mtrigger.com; 4 Dexter Kofa, Mobile App Developer, Philadelphia, PA 19120, USA; dexus@dextech.com; 5 Department of Kinesiology and Applied Physiology, University of Delaware, Newark, DE 19716, USA

**Keywords:** EMG biofeedback, gait, rehabilitation, measurement

## Abstract

Gait impairment often limits physical activity and negatively impacts quality of life. EMG-Biofeedback (EMG-BFB), one of the more effective interventions for improving gait impairment, has been limited to laboratory use due to system costs and technical requirements, and has therefore not been tested on a larger scale. In our research, we aimed to develop and validate a cost-effective, commercially available EMG-BFB device for home- and community-based use. We began by repurposing mTrigger^®^ (mTrigger LLC, Newark, DE, USA), a cost-effective, portable EMG-BFB device, for gait application. This included developing features in the cellphone app such as step feedback, success rate, muscle activity calibration, and cloud integration. Next, we tested the validity and reliability of the mTrigger device in healthy adults by comparing it to a laboratory-grade EMG system. While wearing both devices, 32 adults walked overground and on a treadmill at four speeds (0.3, 0.6, 0.9, and 1.2 m/s). Statistical analysis revealed good to excellent test–retest reliability (r > 0.89) and good to excellent agreement in the detection of steps (ICC > 0.85) at all speeds between two systems for treadmill walking. Our results indicated that mTrigger compared favorably to a laboratory-grade EMG system in the ability to assess muscular activity and to provide biofeedback during walking in healthy adults.

## 1. Introduction

Gait impairment often limits physical activity and negatively impacts the quality of life in individuals with a variety of conditions such as stroke. As such, any interventions that substantially improve gait characteristics also have the potential to increase physical activity. One intervention to improve gait that has been extensively tested within laboratory contexts is electromyography biofeedback (EMG-BFB), a form of neuromuscular biofeedback that uses surface electrodes to detect changes in muscle activity and convey that information back as visual or auditory signals [1]. Although EMG-BFB is the most common physiological variable monitored using biofeedback in research studies [2], researchers have infrequently translated its use from the laboratory to home- or community-based settings. As a measure of change in muscle activity, EMG-BFB can be used in rehabilitation to either increase activity (e.g., in weak or paretic muscles) [3] or decrease activity (e.g., in spastic muscles) [4], or to change activation patterns (e.g., muscle co-activation) [5]. EMG-BFB is effective in both musculoskeletal and neurological rehabilitation [1], and its feasibility has been demonstrated in many diverse contexts such as anterior cruciate ligament reconstruction [6], arthroscopic partial meniscectomy [7], knee osteoarthritis [8], reduction in muscular tension in the neck [9], improving the gait of children with cerebral palsy [10], and improving walking push-off muscle activity in older adults [11]. Additionally, it has been shown that EMG-BFB may be a viable clinical method for targeting corticomotor excitability [12].

Recently, researchers have taken advantage of biofeedback to achieve symmetrical gait patterns in different populations with asymmetrical gait [13]; these interventions appear to result in moderate to large treatment effects immediately after gait retraining [14]. Similar results were found in a systematic review by [15], where a meta-analysis on 11 eligible biofeedback studies (PEDro score > 4, mean 5.7, indicating high-quality trials) indicated that this technique leads to improvements in lower limb activities following stroke. Within these studies, biofeedback increased walking function compared to typical therapy, with a moderate effect size of SMD = 0.57 [15]. These results were replicated in another recent study that compared improvements from EMG biofeedback to conventional physical therapy on gastrocnemius function [3]. This study found association measures with a larger effect size (1.25) than that seen in previous research [15], with improvements in both walking speed and step length resulting from EMG-BFB.

Studies examining EMG-BFB [3,16–23] in stroke gait rehabilitation have reported improved gait speed, step length, ankle power [3,4,5,6,7,8,9,10,11,12,13,14,15,16], muscle strength [17,18,19,20,21], balance [18], higher motivation, reduced spasticity and higher muscle force [19] increased stride length, walking velocity, gait symmetry, and push-off impulse [20] and improved stride length and velocity [22], as well as reduced dependence on assistive devices [23]. These findings suggest that EMG-BFB may be an effective means by which rehabilitation practitioners can improve the characteristics of gait, contingent on the availability of valid and reliable instruments.

The need for valid and reliable EMG BFB devices for clinical and home use was also noted in a recent review published in 2021 on the application of biofeedback for post-stroke gait retraining in the context of emerging technologies [24]. Despite the extant literature showing its effectiveness in improving functional outcomes [3,15,25,26,27,28,29,30,31,32,33,34,35,36,37,38], EMG-BFB has been used almost exclusively within research studies and not as a clinical or field-based tool. One contributing factor has been the technological hurdles related to use outside the laboratory [24]. Laboratory-grade EMG systems are cumbersome, complex, and require significant setup preparation before the application of sensors (including the need for computer and cabling and syncing across devices), followed by the extensive post-processing of signals. All these technical issues limit the use of traditional EMG-BFB systems in field-based community or rehabilitation settings. On top of these technical issues, there are educational and cultural barriers such as the general acceptance of objective measurement and evidence-based medicine, and lack of user-friendly devices for collecting and interpreting EMG data [39]. Compounding this is the high cost of such systems, which places further limits on the use of existing EMG-BFB systems and makes it unpractical for home rehabilitation.

Research on the application of biofeedback in the gait of older adults has shown that this population has an underutilized propulsive reserve that can be exploited by using biofeedback to improve walking function [11]. Additionally, research has indicated that individuals with stroke are less physically active than other individuals [40]. This population would therefore benefit from a home- or community-based EMG-BFB rehabilitation tool to help improve walking characteristics and ultimately increase the number of steps taken on a daily basis. With the predicted stroke population set to increase by almost 25% by 2030 [41], this makes the urgency of translational research even greater [42,43]. Additionally, the COVID-19 pandemic has illustrated the need for alternative, home-based rehabilitation paradigms to be used in tandem with conventional therapy. Thus, translation of an EMG-BFB technology may provide an opportunity to address the rehabilitation requirements of different populations with gait impairment, while at the same time reducing the burden of health costs and on caregivers [44]. Considering the explosion of health-related devices and data, the National Institute of Neurological Disorders and Stroke (NINDS) has designated evaluating the validity and effectiveness of these data sources in providing value to stroke epidemiology and surveillance a major priority. Therefore, we have focused on repurposing and demonstrating the feasibility of mTrigger, a cost-effective, portable EMG-BFB device, as our first step in addressing these challenges and providing both clinicians and patients with a practical device to implement this technique.

**Figure 1:**
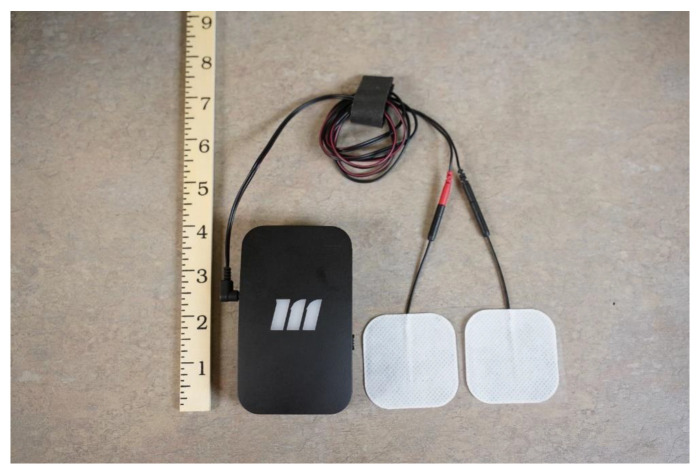
mTrigger device and disposable electrodes. This is a 2-channel system, but in this study, only 1 channel was used.

mTrigger^®^ (https://www.mtrigger.com, accessed on 28 April 2021) is a portable, two-channel, EMG-based biofeedback device that pairs with iPhone or Android cellphones through Bluetooth^®^ technology to provide real-time muscle activity feedback through a mobile application. Originally developed as a tool to improve athletic performance, the mTrigger system provides a unique opportunity to translate laboratory-based EMG-BFB research for more wide-spread clinical or home use. Figure 1.

With this in mind, our research had two overarching goals:

I: Initial testing and repurposing: Before we could test the mTrigger for validity and reliability, we had to test for signal drift and lag during training.

Initial Testing: the mTrigger device would need to have a sufficiently high temporal resolution to capture stride data and exhibit little or no signal drift in order to be appropriate for gait retraining. To test for the temporal lag in plantar flexors activity detection, we hypothesized that mTrigger would detect the same number of EMG bursts of activity when the participants rapidly performed heel rises, resulting in the rapid activation and relaxation of the calf muscles. To test for drift, we hypothesized that the mTrigger signal would not significantly change when participants performed no activity in a seated position.

Repurposing: To repurpose mTrigger for gait application, we added several features:

1- *Auditory and haptic feedback* was added to the settings so that the participant would be able to select the mode of feedback when the activation goal is reached.
2- *Success Rate* was defined as the number of successful steps (where activation goal is met) divided by all steps (where the minimum threshold is met). The threshold can be defined in the settings of the app, but must be lower than the activation goal. The threshold was added to detect the muscle peaks that would correspond to a step that falls short of the activation goal. Success rate was added with motor learning in mind. This acts as both a measure of overall session performance and an outcome measure;
3- *Calibration* was added to detect and display the mean for peak muscle activity during the walking trial. This is beneficial in two ways: it can act as a normalization feature and can act as a baseline to set higher goals of activity for the muscle;
4- *Cloud Upload* was added with home and community training in mind. mTrigger signals from both channels are uploaded to Google Cloud and available for further investigation.

II: Validity and Reliability: Our goal was to establish the validity and reliability of the mTrigger system in the detection of planter flexor muscle activity during push-off at walking speeds typically demonstrated by older adults and individuals with stroke. Gastrocnemius activity has one large peak prior to push-off and, along with the soleus, provides push-off and propels the body forward [45,46]. We hypothesized that the mTrigger system would measure the same number of EMG bursts of activity as a laboratory-grade DELSYS (Natick, MA, USA) EMG system (Bagnoli EMG 2) when participants walked at four different speeds equivalent to and faster than those demonstrated by individuals with gait impairment. We selected these speeds to match household ambulation or severe gait impairment (<0.4 m/s), limited community ambulation (between 0.4 m/s and 0.8 m/s), full community ambulation (>0.8 m/s) as defined by Perry et al. [47], and normal walking speed (1.2 m/s). We also hypothesized that mTrigger would measure the same number of EMG bursts of activity when these participants walked at the same speed in a follow-up trial. We had the same hypothesis regarding the overground walking, comparing the mTrigger system to a metronome as participants matched their steps to metronomes to approximate treadmill walking speeds.

## 2. Materials and Methods

A total of thirty-two participants (*n* = 32, $\overset{-}{x}$ =25.22 ± 5.01 years, 17F/15M) participated in the data collection. All 32 performed the treadmill walking assessment, and of these, 21 performed an additional overground walking trial. Prior to initiating data collection, the research protocol obtained Institutional Review Board approval, and all participants signed informed consent prior to data collection. In order to be included in this study, participants had to be between the ages of 18 and 70 years old and able to walk without assistive devices, and also have no recent musculoskeletal injuries. Exclusion criteria included a history of gait disorders, mild to severe osteoarthritis, and any neurological disorders that affected gait.

### 2.1. Repurposing mTrigger for Gait Rehabilitation

**Figure 2:**
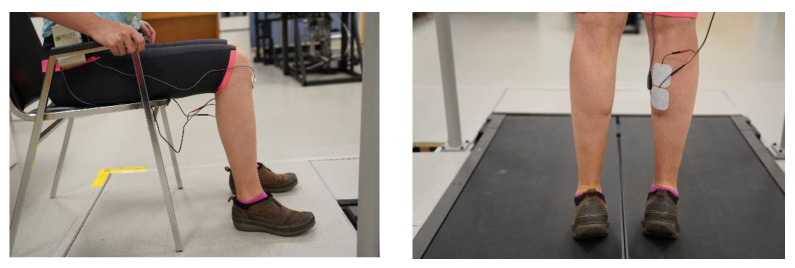
(**Left**): Drift test—participants spent 5 min in a seated position with knees flexed at 90 degrees and were instructed not to move. (**Right**): Lag test—participants performed heel rises to match a metronome at 45, 60, 75, and 90 BPM for 30 s.

Drift: In order to check for drift, EMG and mTrigger data were collected for one block of 5 min while participants maintained a seated position, knees flexed at 90 degrees, with no movements from the medial gastrocnemius. EMG data collection details are explained in further detail in Section 2.2. We used five-minute increments because it is the maximum time that can be set for each block of activity using the mTrigger system. Linear regression was used to fit a line through the data, and the beta values were compared to zero to check if they were significantly different. Figure 2.

Lag: The goal of this test was to determine if the mTrigger system could detect successive contractions and relaxations of plantar flexors that corresponded to push-off phases of the gait. We wanted to determine if the mTrigger system could detect human cadences found in normal and rigorous ambulation (approximately between 100 and 130 steps per minute [48]). These numbers would correspond to 50–65 peaks in EMG activity. Therefore, we tested a range from 45–90 to determine if the mTrigger system would detect faster and slower muscle contractions and relaxations. Participants performed 30 s of standing heel rises at 4 different frequencies while EMG data were recorded. The order of trials was randomized, and participants were instructed to stand still for 15 s before and after each test. Participants performed rapid heel rises to the full range of motion at 45, 60, 75 and, 90 BPM in a standing position. Each trial lasted 30 s and had 15 s of no activity before and after the heel rises. Output measures included the number of peaks detected by the mTrigger and DELSYS systems. Figure 2.

**Figure 3:**
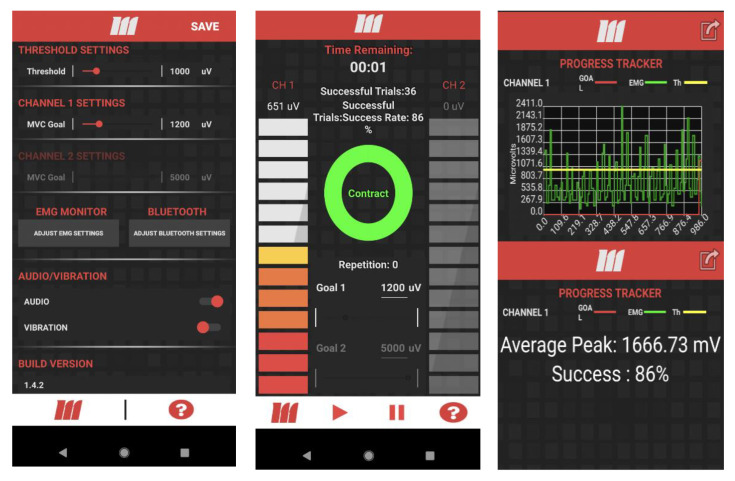
(**Left**) The setting panel for the mTrigger phone app. The threshold and goal are set on this screen. Additionally, audio, and haptic (‘vibration’) feedback can be turned on or turned off. (**Middle**) This panel is displayed during training. Near the top, time remaining as well as the number of successful trials and overall success rate is shown for the trial. The red to gray bar graph on the left side indicates progress towards a force goal for Channel 1. (**Top Right**) This shows the tracking panel. This panel is used to display the session data. The square button with the arrow is used to upload the data to Google Cloud. (**Bottom Right**) This is displayed when clicking on the tracked data (**Top right**), which shows the average peak muscle activity for the duration of trial and the success rate. This average number can be used in an initial trial to detect and set the initial threshold and subsequent activation goals. Success rate can be used as an outcome measure and additionally to make sure participants meet the increased goal at a minimum rate.

Incorporation of haptic and auditory feedback: There were several changes that we deemed necessary for repurposing mTrigger. mTrigger provides visual feedback on muscle activity. For an EMG-BFB device for gait rehabilitation, this feedback is not optimal. Visual information plays an important role in the control and stability of human gait. It improves stability and behavior such as obstacle avoidance and navigation [49]. As such, visual feedback is not preferable for gait because it limits one of the most important tools needed. We added the ability to provide *auditory and haptic biofeedback* to the mTrigger system as an alternative to visual feedback. The addition of haptic feedback is beneficial in several ways. Firstly, it makes training possible for subjects that are hard of hearing or if the auditory feedback is distracting. Secondly, vibrations make devices usable if participants are intending to use the device in an assistive manner or in an environment where the auditory feedback would be distracting to others. Figure 3.

Incorporation of calibration (peak detection and averaging): EMG signals can be highly variable [50]. Some of the factors that lead to these variations are intrinsic, and others are extrinsic [51]. Factors such as electrode placement, configuration, skin preparation and impedance [52], and skin temperature [53] are considered extrinsic, whereas physiological factors include anatomy, biochemical characteristics of the muscles, and blood flow [51]. Crosstalk is another source of error that depends on physiological parameters [54] and electrode size [55], where signals from muscle groups that are not monitored contaminate the signal that is collected. Additionally, the sensor skin interface, motion artifacts, and deformation of this interface are other sources of error in wearable bioelectric signal monitoring [56]. These characteristics can vary in an individual between days or even within a day if placement changes [51]. Moreover, when a device is used as a rehabilitation tool, it is expected that both intrinsic and extrinsic factors will change. Although these sources of error and artifacts cannot be eliminated, the addition of a normalization or calibration feature can help to reduce the variability associated with some of them. For this reason, we added a *calibration feature* to the app. When determining the threshold for feedback levels, the app calculated the mean peak activity level for the muscle during the calibration movement when the user clicked on the collected data. To calculate the peak mean activity, we applied a previously published peak detection algorithm [57] on the mTrigger signal. We discarded the first 10 s of data and discarded all the data below the threshold. Then, we used the algorithm to calculate a moving mean and standard deviation based on 5 time points and 3.5 standard deviations to calculate the number of peaks and find the mean peak muscle activity. In the case of gait, we used 1 min of normal walking to determine the average peak muscle activity to be used as a baseline for setting activation goals. Figure 3.

Incorporation of cloud upload: *Cloud upload* was added with home training in mind. After each session, participants were asked if they wanted to upload the data to the cloud. These data were then available through Google Cloud to the researcher. Additionally, cloud integration makes of further epidemiological studies in gait rehabilitation possible, which is of interest to NINDS [44]. Figure 3.

Incorporation of success rate: A robust finding in motor learning literature is the critical role of providing feedback during the practice. Feedback during the practice can be in the form of knowledge of performance (KP) or knowledge of results (KR). KR usually refers to feedback about achieving the goal or not, whereas KP refers to specific characteristics of the movement [58,59,60]. Both types of feedback are effective in improving motor learning and have been utilized in stroke rehabilitation [61,62]. In order to utilize both KR and KP, we added a new feature to mTrigger to provide participants with a measure of success that takes the whole trial into account. We added a second threshold to the app to define success rate, which is also displayed in the app. *Success rate* was defined as the number of successful steps (when the participants meet activation goal) divided by all the steps (when the participants meet the threshold). The threshold is used to calculate all the steps (activation which meets the criteria for being a step but does not reach the goal). Addition of a success rate made both KP and KR available, provided an overall performance measure for the duration of practice session, and could be used to optimize the practice session or set goals for rehabilitation such as achieving at least an 80 percent success rate at a determined goal level of 20 percent or 40 percent above mean peak muscle activity. Figure 3.

### 2.2. Validity and Reliability

**Figure 4:**
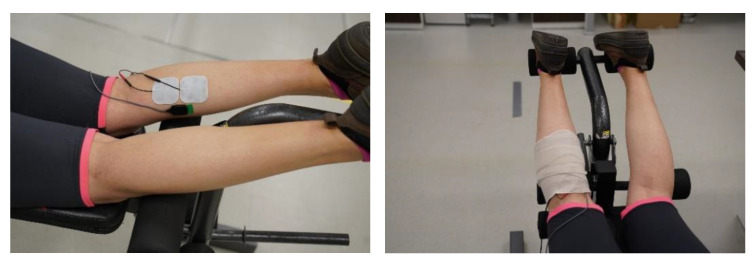

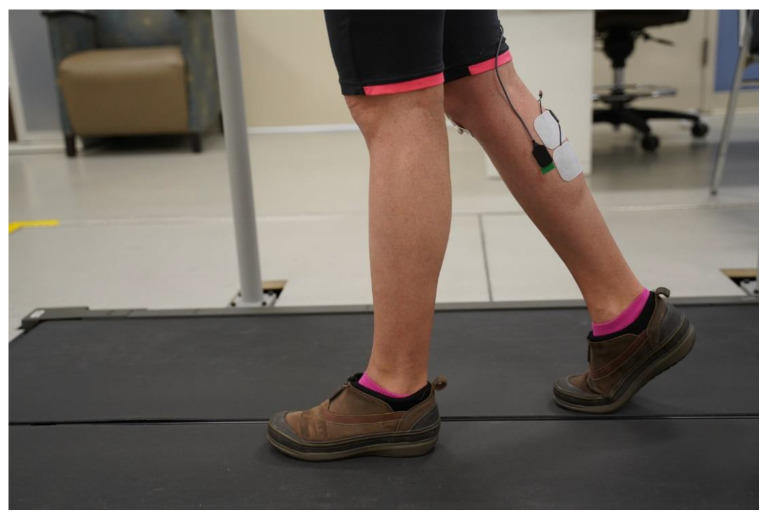
(**Top Left**) mTrigger and DELSYS electrode placement. Electrodes were placed on the medial gastrocnemius with DELSYS electrode always medial to mTrigger electrodes. (**Top Right**) A flex wrap was used to minimize electrode movement and wired movement. Wraps were removed to illustrate electrodes in different positions and different data collection settings. (**Bottom**) Participant in a push-off position walking on a treadmill.

Participants had the skin on top of the plantar flexors on both sides of the body prepared prior to data collection in order to optimize signals. We shaved excess hair and then cleaned the skin with alcohol. Next, both mTrigger and DELSYS electrodes were attached to the medial gastrocnemius per Surface Electromyography for the Non-Invasive Assessment of Muscles (SENIAM) recommendations. This procedure was repeated in the second data collection session. The medial gastrocnemius was identified through palpation. Both mTrigger electrodes and DELSYS electrodes were placed on the muscle belly (the DELSYS electrode was always medial but not touching two mTrigger electrodes) and secured with flex wraps. The mTrigger device was paired with an android cellphone (Motorola Moto G4 (Motorola Mobility, Chicago, IL, USA, 2016), Google Pixel 4a (Google, Mountain View, CA, USA, 2020)) via Bluetooth^®^, and EMG data from the DELSYS system were collected using a custom LabVIEW program. Figure 4.

Connectivity to LabVIEW and mTrigger was checked prior to data collection by asking participants to perform a heel rise. Then, the activation goal in the mTrigger app was calculated from an initial trial where participants walked at each speed (0.3, 0.6, 0.9, 1.2 m/s) for 1 min. This was made possible by the calibration feature that we added, and calculated the mean peak average muscle activity. This value was then used as the EMG goal, and the minimum threshold was set at 20 percent below this value, meaning that the algorithm would detect a burst of activity as a step if the signal peaked above this threshold. The DELSYS and mTrigger systems were manually synced because it was not possible to externally trigger the mTrigger app.

Prior to the beginning of the data collection, participants walked for 5 min to stabilize their gait. Next, walking data were collected at 4 speeds (0.3 m/s, 0.6 m/s, 0.9 m/s 1.2 m/s) in two separate trials for 2 min each. These speeds were chosen as representative speeds corresponding to household ambulation or severe gait impairment (<0.4 m/s), limited community ambulation (between 0.4 m/s and 0.8 m/s), full community ambulation (>0.8 m/s) in stroke gait as defined by Perry et al. [47], and normal walking speed (1.2 m/s). Electrodes were removed and attached again for the second trial. The second trial either took place in the same data collection session or within one week, based on subject’s schedule. The order of trials was randomized, and participants rested between trials. The numbers of peaks per trial, corresponding to the number of right limb steps, were compared between DELSYS and mTrigger.

For overground walking, the number of steps was counted for one minute while participants were completing their treadmill walking to determine the number of steps they took per minute. This value was then used to pace their overground walking using a quartz metronome device (SEIKO SQ44, Tokyo, Japan). Participants were instructed to walk continuously back and forth along a 10 m pathway while matching their steps to the metronome beat.

The mTrigger system is designed to provide biofeedback that approximates muscle activity, and as such, has signal processing steps that are distinct from standard EMG processing. Per communication with the manufacturer, signal processing steps included a first-order low pass passive filter, second-order active bandpass filter, and first-order low pass passive filter. To determine if the two devices provided similar biofeedback from the gastrocnemius during push-off, the number of EMG peaks during walking trials was compared between the DELSYS and mTrigger systems to establish validity. Raw EMG data collected by the DELSYS system were imported to MATLAB (Version 2020b, MathWorks, Inc., Natick, MA, USA), processed, and a custom code was written to detect the number of peaks. The processing steps in the order of application were demeaning the data, bandpass filtering (20–500 Hz) using a sixth-order Butterworth filter, full-wave rectification, and applying a fourth-order low pass Butterworth filter (10 Hz) to smooth the signal.

### 2.3. Statistical Analyses

The required number of participants to obtain sufficient statistical power for performing validity analysis between mTrigger and DELSYS was calculated to be *n* = 14 [63]. This analysis was performed at the α = 0.05 significance level powered at 1-β = 0.80 with minimum acceptable reliability (ICC) = of 0.6 and expected reliability of (ICC) = 0.9.

We completed all analyses using IBM SPSS Statistics for Windows, version 27 (IBM Corp., Armonk, NY, USA). To test for drift, we used linear regression to fit a line to resting mTrigger data; then, we used a two-tailed single-sample *t*-test to test whether the beta value was significantly different from zero. To test for temporal lag, Pearson’s correlation was used between the number of peak contractions of the right limb gastrocnemius as detected by DELSYS and mTrigger.

To establish the validity of the mTrigger system compared to the DELSYS system while walking on the treadmill, a single measure intraclass correlation with mixed effects (consistency) was performed (α = 0.05). To establish validity of the mTrigger system compared to the metronome during the overground walking, we also performed a single measure intraclass correlation with mixed effects (consistency) performed at (α = 0.05). In all instances, the dependent variable was the number of right footsteps for the duration of the trial. ICC results were reported based on the following range as moderate (0.5–0.75, *), good (0.75–0.90, **), or excellent (>0.90, ***) [64].

Reliability was measured using Pearson’s correlation coefficient. To establish reliability, we examined mTrigger data between two separate testing sessions. In all instances, the dependent variable was the number of right footsteps for the duration of the trial. Correlation coefficient results were reported based on following range as moderate (0.50–0.70, +), high (0.70–0.90, ++), or very high (>0.90, +++) [65].

## 3. Results

### 3.1. Drift

In order to assess drift, we used a linear regression model (Equation (1)) to fit the data, and then we used a two-tailed single-sample *t*-test to see if β1 was significantly different from zero. (β0 = 84.23 ± 32.98, β1 = 0.000137 ± 0.0018). Results were not significantly different from zero, with t = −0.000001, *p* = 0.99, indicating no significant signal drift.

y = β0 + β1x(1)

Additionally, during pilot data collection, we noticed sudden, high-frequency bursts of activity when collecting data for drift. To address this issue, a low pass filter was applied inside the mTrigger app which successfully removed these high-amplitude random bursts.

### 3.2. Temporal Lag

For all the participants, the number of peaks detected by the mTrigger system matched those detected by the DELSYS system. As a result, the Pearson’s correlation coefficient between mTrigger and DELSYS for detection of heel rise was *r* = 1.00, *n* = 31, *p* < 0.0001. These data indicate that any temporal lag of mTrigger will not result in a missed step (45 < (50–65) < 90) [48].

### 3.3. Validity: Treadmill Walking

**Figure 5:**
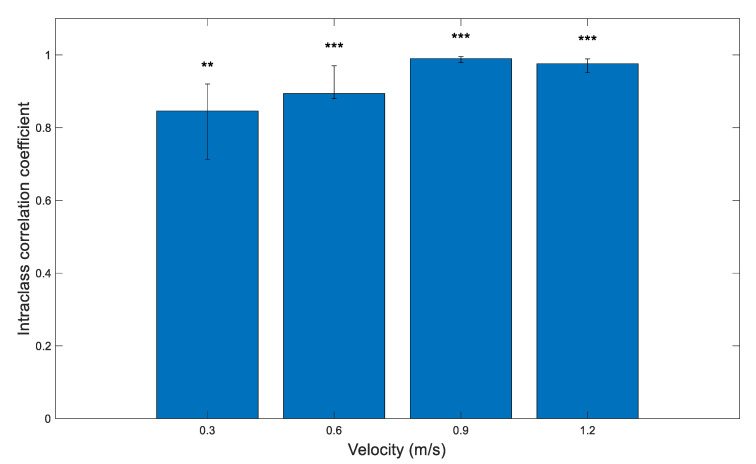
Single measure intraclass correlation coefficients (ICC) between right limb EMG peaks of the medial gastrocnemius detected by mTrigger and DELSYS at different speeds for treadmill walking with 95% confidence interval. All correlations were significant at *p* < 0.05. Agreement: ** = good, *** = excellent.

Single measure intraclass correlation with two-way mixed effects (*p* < 0.001) revealed good level of agreement between number of peaks detected by the mTrigger and DELSYS systems during walking at household ambulation speed (0.3 m/s), and excellent agreement at speeds of limited community ambulation (0.6 m/s), full community ambulation (0.9 m/s) and normal walking (1.2 m/s). The results are presented in Figure 5 and Table 1.

### 3.4. Validity: Overground Walking

**Figure 6:**
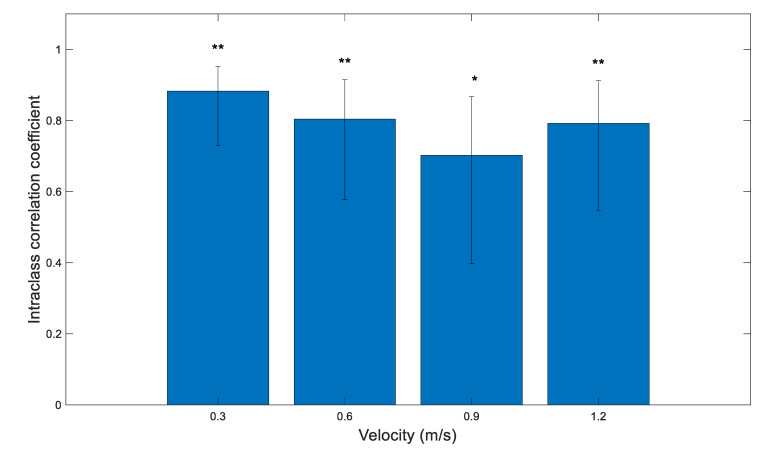
Single measure intraclass correlation coefficients (ICC) between the number of right limb EMG peaks of the medial gastrocnemius detected by mTrigger and DELSYS at different speeds for overground walking with 95% confidence interval. All correlations were significant at *p* < 0.05. Agreement: * = moderate, ** = good.

Single measure intraclass correlation (*p* < 0.001) revealed a good level of agreement between the number of peaks detected by the mTrigger and DELSYS systems during walking speeds for household ambulation (0.3 m/s), limited community ambulation (0.6 m/s), and normal walking speed (1.2 m/s). There was moderate agreement at full community ambulation speed (0.9 m/s). The results are presented in Figure 6 and Table 2.

### 3.5. Reliability: Treadmill Walking

**Figure 7:**
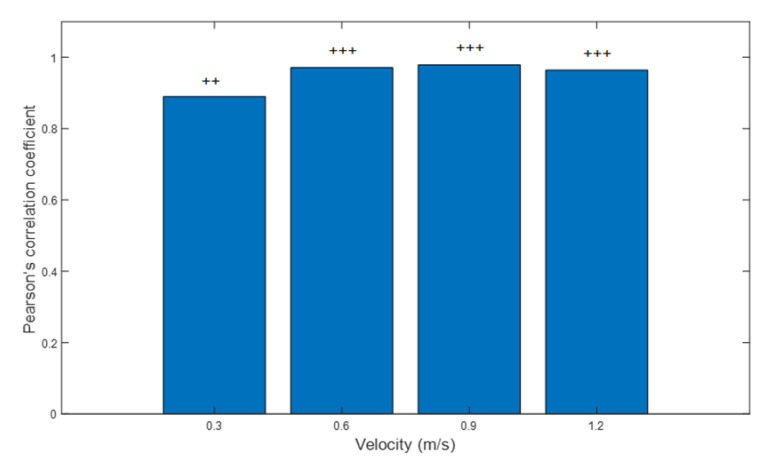
Pearson’s correlation between the number of right limb EMG peaks of medial gastrocnemius activity between two sessions of treadmill walking. Overall results indicate a good to excellent level of agreement All correlations were significant at *p* < 0.05. Correlation: ++ = high, +++ = very high.

Significant Pearson’s correlation coefficients (*p* < 0.001) revealed high or very high positive correlation between the number of peaks detected by mTrigger between session 1 and session 2. Results indicated very high positive correlation at community ambulation speed (0.6 m/s, CC = 0.971), full ambulation speed (0.9 m/s, CC = 0.979) and normal walking speed (1.2 m/s, CC = 0.964). There was also a high positive correlation at limited ambulation speed (0.3 m/s, CC = 0.890). The results are presented in Figure 7.

### 3.6. Reliability: Overground Walking

**Figure 8:**
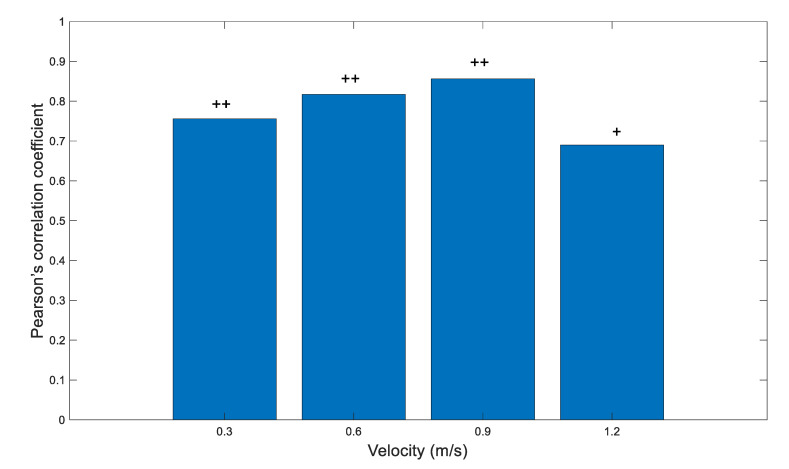
Pearson’s correlation between the number of right limb EMG peaks of medial gastrocnemius activity between two sessions of overground walking. Overall results indicate moderate to good level of agreement. All correlations were significant at *p* < 0.05. Correlation: + = moderate, ++ = high.

Significant Pearson’s correlation coefficients (*p* < 0.001) revealed a moderate to high correlation between the number of peaks detected by mTrigger between session 1 and session 2. Results indicated high positive correlation at limited ambulation speed (0.3 m/s, CC = 0.756), community ambulation speed (0.6 m/s, CC = 0.817), and full ambulation speed (0.9 m/s, CC = 0.856), and moderate positive correlation at normal walking speed (1.2 m/s, CC = 0.690). The results are presented in Figure 8.

## 4. Discussion

The current research had two main purposes, with the overarching goal of determining the suitability of the mTrigger system as a valid, reliable method of providing EMG-BFB. First, we tested the mTrigger device for drift and lag, then repurposed it so that it could be used in clinics or home- and community-based settings. We found that the device did not show a significant drift during a 5 min resting task and that any temporal lag would not result in missed step detection. Next, we compared the mTrigger system to DELSYS, a laboratory-grade EMG system. We hypothesized that the mTrigger system would detect EMG bursts of activity during rigorous muscle contraction–relaxation, similarly to the DELSYS system. This hypothesis was upheld. Next, we hypothesized that the mTrigger system would detect the same number of EMG bursts of activity when participants rapidly performed heel rises: this hypothesis was upheld as well. The mTrigger device correctly captured maximum plantar flexor muscle contractions at rates similar to and higher than those typically presented by individuals with stroke or older adults. This suggests that mTrigger feedback provides near-real-time feedback for gait applications. Additionally, we developed specific features in the mTrigger device to make it suitable for gait application. This included adding both auditory and haptic feedback, a calibration/normalization feature, success rate output, and a cloud upload feature.

Once we repurposed the device to be used in gait research, the second step was to establish the validity and reliability of the mTrigger system in detecting gastrocnemius activity during active walking at speeds comparable to those of older adults and individuals with gait disorders by comparing it to the DELSYS system.

Finally, we hypothesized that the mTrigger system would correctly measure the same number of peaks in the medial gastrocnemius (which corresponds to push-off phase and is often the target of biofeedback trainings) as the lab-grade DELSYS system, also demonstrating the validity of the mTrigger signal. This hypothesis was upheld, and our results showed good to excellent intraclass correlation between mTrigger and DELSYS (0.990 > ICC > 0.846) for treadmill walking.

Our data showed moderate to good intraclass correlation between mTrigger and DELSYS (0.883 > ICC > 0.702) for overground walking. The fact that these ICCs were lower than those found in treadmill walking was most likely caused by the walking path used in our overground data collection. Due to the room dimensions, participants had to perform a U-turn at both ends of the 10 m pathway. Interestingly, the lowest ICC was achieved during slow walking on a treadmill (0.846); by comparison, this was the highest ICC in the overground conditions (0.883). One possible explanation for this disparity may be the fact that because participants were walking more slowly over the ground, the sudden changes when making U turns and subsequent changes in muscle activity were reduced, both in the number of turns and magnitude of change during the slower condition.

After establishing its validity, we tested the reliability of the mTrigger system. We hypothesized that the mTrigger system would measure the same number of EMG bursts of activity when participants walked at the same speed in two different trials, demonstrating the reliability of the system. This hypothesis was upheld, with very high correlation between two sessions of data collection

We wanted to demonstrate the validity and reliability of the device before further testing with individuals with stroke or older adults; therefore, we collected data at speeds (0.3 m/s, 0.6 m/s, 0.9 m/s and, 1.2 m/s) corresponding to household ambulation, limited community ambulation, and full community ambulation, using normal walking speed as an upper bound. We selected these speeds because they represent the range of our target populations who would benefit from push-off gait biofeedback (e.g., individuals with stroke and older adults). The goal of this research protocol was to modify the mTrigger device for gait rehabilitation, ensure that there was no significant lag or drift, and add features for future home-based training applications. Additionally, we wanted to determine if the modified mTrigger system is a valid and reliable device that could be used as a low-cost alternative to laboratory-grade EMG system. We believe our results support the goals we had envisioned.

## 5. Limitations

Our goal was to establish that the mTrigger system validly and reliably measured gastrocnemius activation that occurred during walking. For this reason, we did not compare the signal characteristics of the DELSYS EMG device with mTrigger signals. Due to DELSYS system wiring limitations, we could not collect overground data along a straight line of more than 10 m; participants had to walk back and forth on a path. This may have resulted in the lower ICC values seen in the overground walking conditions.

Although the mTrigger device was modified with the patient population and home use in mind, the research itself was conducted in a laboratory and on healthy participants in order to establish initial validity and reliability. Therefore, the current study does not contain applied data. For example, we did not have our participants apply the EMG sensor pads to themselves, which is a potential source of signal error, and which should be field-tested in the future. This study was conducted in a laboratory with trained researchers applying sensors in accordance with SENIAM guidelines. It is unrealistic to expect that in a home-based scenario these guidelines can be followed accurately without training. Therefore, researchers interested in home applications of the device will need to field test appropriate educational methods and best practices to ensure fidelity of the mTrigger system in a home setting. It may also be possible to provide software solutions such as augmented reality. Technologies such as LiDAR (light detection and ranging) and TrueDepth [66], have become available in the latest iPhone and Android phones and tablets; therefore, it may be possible to overlay digital information on limbs and provide more accurate sensor placement instructions for home use.

## 6. Conclusions and Future Directions

In conclusion, we found that the mTrigger system, a low-cost, commercially available EMG-BFB device, detected signals similarly to the DELSYS system, a laboratory-grade EMG system during treadmill and overground walking at speeds normally presented by individuals with stroke or older adults. These results suggest that the mTrigger system may be a viable alternative to deliver EMG-BFB in gait rehabilitation, ultimately helping individuals with gait impairment increase their daily physical activity by improving walking characteristics. Translation of EMG-BFB, a successful therapeutic paradigm in stroke [3] and other populations such as knee OA [8] and elderly adults [67], from a research-only methodology to a valid, reliable and accessible technology, is an important step that will complement clinic training and impact home-based training. With the emergence of COVID-19, social distancing and stay-at-home orders, effective home-based training has been and will become more important. Our results suggest that mTrigger, as a low-cost, portable, valid, and reliable EMG-BFB device, holds promises as a means to address this need.

Now that the validity and reliability of this device has been established, careful studies must be conducted to test the biomechanical and neural effects of EMG-BFB using this device on gait, first on healthy participants and then on patient populations. Additionally, patient characteristics must be identified that would best respond to EMG-BFB gait training that targets plantar flexors. Moreover, further studies should be conducted to evaluate the assistive vs. rehabilitative capabilities of the device. Studies on optimal levels of feedback and required dosage should also be conducted to establish guidelines for activation goals and feedback levels based on baseline performance. Finally, larger scale studies can be performed on the utility of EMG-BFB for gait rehabilitation in clinical, home-, and community-based settings.

Our results were very promising; therefore, we now plan to replicate this research on older adults and a sample of individuals with stroke to determine the efficacy of the mTrigger system as a rehabilitation intervention. Although there have been over 1000 randomized clinical trials in stroke rehabilitation, to date, little translation to clinical practice has taken place [68,69]. Therefore, more research must be performed that translates laboratory-based results into applied rehabilitation settings, using wearable technologies such as the mTrigger system that are both cost-effective and commercially available [24]. Our short-term goals are to field test the mTrigger systems in stroke and other neurological gait conditions to assess the immediate effects on improving walking function and walking biomechanics. In addition, we want to optimize the level of feedback (EMG activation goal) to maximize motor learning and facilitate long-term effects through application of the challenge point framework (CPF) [70].

## Figures and Tables

**Table 1:** Intraclass correlation coefficients between medial gastrocnemius peaks detected by mTrigger and DELSYS for treadmill walking at various walking speeds.

Velocity(m/s)	ICC	Confidence Interval
0.3	0.846	*n* = 32, 95% CI (0.713–0.920)
0.6	0.939	*n* = 32, 95% CI (0.880–0.970)
0.9	0.990	*n* = 32, 95% CI (0.979–0.995)
1.2	0.976	*n* = 31, 95% CI (0.951–0.989)

**Table 2:** Intraclass correlation between medial gastrocnemius peaks detected by mTrigger and DELSYS for overground walking at various walking speeds.

Velocity (m/s)	ICC	Confidence Interval
0.3	0.883	*n* = 21, 95% CI (0.729–0.952)
0.6	0.804	*n* = 21, 95% CI (0.577–0.915)
0.9	0.702	*n* = 21, 95% CI (0.397–0.867)
1.2	0.792	*n* = 20, 95% CI (0.547–0.912)

## Data Availability

Data will be made available through this link: https://drive.google.com/drive/folders/12poBL5u6ZSei12Y3vqlPoSPfQ-oJxjeJ?usp=sharing (accessed on 28 April 2021).
